# Predictors of Follow-Up Appointment No-Shows Before and During COVID Among Adults with Type 2 Diabetes

**DOI:** 10.1089/tmj.2022.0377

**Published:** 2023-06-01

**Authors:** Chun-An Sun, Nancy Perrin, Nisa Maruthur, Susan Renda, Scott Levin, Hae-Ra Han

**Affiliations:** ^1^ Johns Hopkins School of Nursing, Baltimore, Maryland, USA.; ^2^ Division of General Internal Medicine, Johns Hopkins School of Medicine, Baltimore, Maryland, USA.; ^3^ Department of Epidemiology, Johns Hopkins Bloomberg School of Public Health, Baltimore, Maryland, USA.; ^4^ Department of Emergency Medicine, Johns Hopkins School of Medicine, Baltimore, Maryland, USA.; ^5^ Department of Health, Behavior, and Society, Johns Hopkins Bloomberg School of Public Health, Baltimore, Maryland, USA.

**Keywords:** appointment no-show, type 2 diabetes mellitus care, telehealth, COVID, telemedicine

## Abstract

**Background:**

The coronavirus disease 2019 (COVID-19) pandemic has rapidly transformed health care delivery into telehealth visits. Attending regular medical appointments are critical to prevent or delay diabetes-related complications. Although telehealth visits have addressed some barriers to in-person visits, appointment no-shows are still noted in the telehealth setting. It is not completely clear how the predictors of appointment no-shows differ between in-person and telehealth visits in diabetes care.

**Objective:**

This retrospective study examined if predictors of appointment no-shows differ (1) between pre-COVID (January 1, 2019–March 22, 2020) and COVID (March 23, 2020–December 31, 2020) periods and (2) by health care delivery modes (in-person or telehealth visits) during COVID among adults with type 2 diabetes mellitus (T2DM).

**Methods:**

We used electronic health records between January 1, 2019 and December 31, 2020 across four diabetes clinics in a tertiary academic hospital in Baltimore, Maryland. Appointments marked as completed or no-show by established adults with T2DM were included in the analyses.

**Results:**

Among 7,276 appointments made by 2,235 patients, overall appointment no-show was 14.99%. Being older and White were protective against appointment no-shows in both unadjusted and adjusted models during both time periods. The interaction terms of COVID periods (i.e., pre-COVID vs. COVID) were significant for when glycated hemoglobin drawn before this visit and for missing body mass index. Telehealth visits during COVID decreased more half of the odds of appointment no-shows.

**Conclusions:**

In the context of diabetes care, the implementation of telehealth reduced appointment no-shows. Future studies are needed to address social determinants of health, including access to internet access, to further reduce health disparities among adults with T2DM.

## Introduction

The coronavirus disease 2019 (COVID-19) pandemic has rapidly transformed health care delivery. Since the Centers for Medicare and Medicaid Services expanded telehealth coverage for all Medicare patients, and for the duration of the COVID-19 Public Health Emergency,^1^ telehealth visits using phone or video conferencing have been used widely in outpatient settings in the United States,^2^ including diabetes clinics. Telehealth is defined as the use of electronic information and telecommunication technologies to provide health care remotely.^3^ The implementation of telehealth visits ensures the continuity of diabetes care when social distancing is required to mitigate the spread of COVID-19.^4^

Type 2 diabetes mellitus (T2DM), accounting for 90–95% of all diabetes mellitus cases,^5^ is a complex and chronic illness that requires active patient engagement in care.^6,7^ To prevent or delay serious diabetes-related complications, American Diabetes Association (ADA) recommends that people with T2DM attend regular medical appointments to evaluate glycated hemoglobin (HbA1c) every 3 months^8^ and to assess vascular complications annually,^9^ on top of actively engaging in lifestyle management.^7,10^ During a high-quality patient-centered diabetes appointment, health care teams support and enhance patient engagement in diabetes care for optimal glycemic control, while people with T2DM receive tailored education and treatment plan.^8^ Yet, studies show that 12–36% of adults with T2DM missed their regular in-person medical appointments pre-COVID.^11,12^

Missed regular appointments among adults with T2DM directly results in poor glycemic control^13^ and has been associated with increased risk for rehospitalization.^14^ Also, appointment no-shows pose substantial financial burdens for providers and health care systems,^15–17^ disturbance of operations, as well as longer waitlists for other patients to seek care.^15,17^ A recent systematic review of 18 studies identified various individual, health care provider and system, and interpersonal factors associated with appointment no-shows among adults with T2DM in the context of in-person visits.^18^

Telehealth visits have addressed some barriers to “in-person” appointments (e.g., transportation, time, and work commitment)^19^ and decreased appointment no-shows.^20^ Nevertheless, appointment no-shows are still noted in the telehealth setting.^21,22^ It is not completely clear why appointment no-shows occur in the telehealth setting and how the predictors of appointment no-shows differ from in-person appointments. Using electronic health record (EHR) data, this study examined (1) if predictors of appointment no-shows differ by pre-COVID versus COVID among adults with T2DM and (2) if predictors of appointment no-shows differ for health care delivery modes (in-person or telehealth visits) among adults with T2DM during COVID.

## Materials and Methods

### STUDY DESIGN OVERVIEW AND SAMPLE

This retrospective study examined predictors of appointment no-shows using scheduled visits from adults (18+ years) with T2DM with at least one prior visit seen at four outpatient diabetes clinics between January 1, 2019 and December 31, 2020 in a tertiary academic hospital in Baltimore, Maryland. The study period was chosen due to the availability of EHR at the time of designing this study in early 2021. Only patients with a zip code within Maryland were included as reimbursement criteria for telemedicine visits differ across states in different time points.

There were no telehealth visits before COVID. All appointments at the clinics were shifted to telehealth visits starting March 23, 2020. Around September 2020, the clinics started scheduling for both in-person and telehealth visits based on patients' preferences and their COVID-19 risk.

**Fig. 1.**
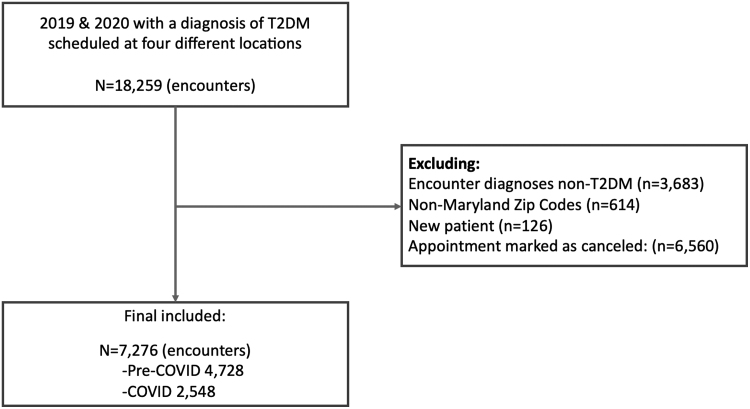
Flow chart of appointment selection. COVID, coronavirus disease; T2DM, type 2 diabetes mellitus.

Our initial EHR inquiry resulted in 18,259 appointments made by 3,742 unique patients. We then excluded 10,983 of those appointments based on the inclusion criteria. Those visits marked as new patient visits were excluded as the reasons for appointment no-shows might differ between new patients versus established patients.^21^ The final cohort included 7,276 appointments (4,728 appointments during pre-COVID [all in-person visits] and 2,548 appointments during COVID [79.36% telehealth visits]) made by 2,235 unique patients (*Fig. 1*). Appointment no-show rate was 17.41% (823/4,728) during pre-COVID and 10.52% (268/2,548) during COVID, with an overall appointment no-show of 14.99%.

### DATA SOURCES

All data were captured by EHR. The Johns Hopkins Medicine Institutional Review Board approved this study design and procedures (IRB00231790).

### STUDY VARIABLES AND OUTCOME REVIEW

#### Outcome

Appointment status was coded as completed or no show.

#### Predictor variables

Literature review guided the variables chosen.^18^ *Supplementary Table S1* shows the details of variables and their operational definitions. Covariates included basic characteristics (age, sex, race, and ethnicity). Age was categorized as <46, 46–60, 61–75, or >75 years old to create equal groups.

Additionally, we included the following factors: T2DM-related and comorbidity, health utilization, health behaviors, and others. Specifically, T2DM-related and comorbidity factors included: HbA1c associated with this visit, when associated HbA1c was drawn before this visit, body mass index (BMI) (missing, normal [≤24.9], Overweight, Class 1, Class 2, or Class 3 obesity^23^), diabetes complications (yes, no), types of T2DM medications (no medications on file, metformin, other than metformin but no insulin, any insulin), depression, or other comorbidities (count of hypertension, hyperlipidemia, coronary heart disease, heart failure, chronic kidney disease, stroke, cancer, Chronic Obstructive Pulmonary Disease emphysema, dementia, HIV/AIDS, liver disease, other advanced illness^24,25^).

Health utilization factors included: emergency department (ED) or hospitalization 12 months before this visit, and patient portal status. Health behaviors included: smoking status, alcohol status, and recreational drug status. Other variables related to social determinants of health (SDOHs) were also included: marital status, selected preferred language on file, emergency contact on file, distance between home's zip code and clinic's zip code, and insurance type.

Finally, we also included health care provider factors (types of providers), health care system factors (scheduling lag, health care delivery modes), and COVID time periods.

### STATISTICAL ANALYSIS

To examine if predictors of appointment no-shows differ by time periods (i.e., pre-COVID vs. COVID), the association of each predictor with appointment no-show and the interaction of each predictor and time period were assessed using random effects logistic regression with visits nested within patients. All variables significant at the *p* < 0.05 level were then entered into a multivariable random effects logistic regression to examine the relative importance of the factors in predicting appoint no-shows.

To examine if the predictors of no-show differ for health care delivery modes (in-person vs. telehealth) during the COVID, the interaction of the health care delivery mode with each predictor were tested separately with random effects logistic regression. To facilitate interpretation of the interactions and examine difference in effect sizes, stratified analyses by health care delivery mode are presented. All statistical analyses were conducted in Stata 17.0.^26^

### POWER ANALYSIS

In this sample, there were 4,728 encounters from 1,913 people during pre-COVID and 2,548 encounters from 1,424 people during COVID. Intraclass Correlation Coefficient (ICC) estimated from the data was 0.36 during pre-COVID and 0.18 during COVID. Our power analysis estimated the odds ratios (ORs) detectable accounting for the ICC with power = 0.80 and α = 0.05, varying the distribution of the predictors, we can detect significant ORs ranging from 1.29 to 1.50 for pre-COVID and 1.48–1.83 for COVID.

## RESULTS

### CHARACTERISTICS OF STUDY SUBJECTS

*Table 1* shows the distribution of patient-level (fixed input throughout different encounters) and encounter-level (might be different across encounters for a patient) variables.

**Table 1.** The Distribution of Predictors Between Pre-COVID and COVID Period

PATIENT-LEVEL VARIABLES			
	OVERALL (***N*** = 2,235)	PRE-COVID (***n*** = 1,913)	COVID (***n*** = 1,424)
	COUNT (%)	COUNT (%)	COUNT (%)
Female	1,107 (49.53)	935 (48.88)	703 (49.37)
Race			
Black	1,089 (48.72)	928 (48.51)	691 (48.53)
White	915 (40.94)	785 (41.04)	592 (41.57)
Others	231 (10.34)	200 (10.45)	141 (9.90)
Hispanic	76 (3.40)	66 (3.45)	49 (3.44)
Patient portal account activated	1,875 (83.89)	1,587 (82.96)	1,253 (87.99)
Marital status as married	1,019 (45.6)	1,049 (55.36)	797 (55.97)
Family member as emergency contact	2,049 (91.68)	1,760 (92.00)	1,309 (91.92)
Home zip code to clinic ≤30 miles	1,895 (84.79)	1,628 (85.10)	1,205 (84.62)

**ENCOUNTER-LEVEL VARIABLES**			
	**Overall (*N* = 7,276)**	**Pre-COVID (*n* = 4,728)**	**COVID (*n* = 2,548)**
	**Count (%)**	**Count (%)**	**Count (%)**
Appointment no-show	1,091 (14.99)	823 (17.41)	268 (10.52)
Age in category, years old			
<46	848 (11.65)	546 (11.55)	302 (11.85)
46–60	2,513 (34.54)	1,646 (34.81)	867 (34.03)
61–75	3,152 (43.32)	2,056 (43.49)	1,096 (43.01)
>75	763 (10.49)	480 (10.15)	283 (11.11)
HbA1c			
Controlled (≤7.0%)	2,585 (35.53)	1,643 (34.75)	942 (36.97)
Uncontrolled (>7.0%)	3,698 (50.82)	2,426 (51.31)	1,272 (49.92)
Missing	993 (13.65)	659 (13.94)	334 (13.11)
When HbA1c drawn before current appointment, months			
Missing	993 (13.65)	659 (13.94)	334 (13.11)
0–3	2,838 (39.00)	1,860 (39.34)	978 (38.38)
4–6	2,081 (28.60)	1,400 (29.61)	681 (26.73)
7–9	973 (13.37)	582 (12.31)	391 (15.35)
10–12	391 (5.37)	227 (4.80)	164 (6.44)
BMI category			
Under or normal weight (BMI ≤24.9)	849 (11.67)	529 (11.19)	320 (12.56)
Overweight	1,945 (26.73)	1,324 (28.00)	621 (24.37)
Class 1 obesity	1,901 (26.13)	1,247 (26.37)	654 (25.67)
Class 2 obesity	1,343 (18.46)	889 (18.80)	454 (17.82)
Class 3 obesity	1,099 (15.10)	719 (15.21)	380 (14.91)
Missing	139 (1.91)	20 (0.42)	119 (4.67)
Medication			
No medication	570 (7.83)	281 (5.94)	289 (11.34)
Metformin only	313 (4.30)	232 (4.91)	81 (3.18)
More than metformin but no insulin	981 (13.48)	673 (14.23)	308 (12.09)
Any insulin	5,412 (74.38)	3,542 (74.92)	1,870 (73.39)
Had an ICD code with diabetes complication	6,600 (90.71)	4,299 (90.93)	2,301 (90.31)
Had ED visit(s) in prior 12 months	1,733 (23.82)	1,141 (24.13)	592 (23.23)
Had hospitalization(s) in prior 12 months	1,486 (20.42)	952 (20.14)	534 (20.96)
Smoking status			
Active	653 (8.97)	412 (8.71)	241 (9.46)
Never	3,959 (54.41)	2,592 (54.82)	1,367 (53.65)
Quit	2,563 (35.23)	1,696 (35.87)	876 (34.03)
Not asked, missing	101 (1.39)	28 (0.59)	73 (2.86)
Alcohol status			
Active	2,595 (35.70)	1,727 (36.53)	868 (34.07)
Never	3,980 (54.70)	2,659 (56.24)	1,321 (51.84)
Quit	356 (4.89)	137 (2.90)	219 (8.59)
Not asked, missing	345 (4.74)	205 (4.34)	140 (5.49)
Recreational drug status			
Active	289 (3.97)	181 (3.83)	108 (4.24)
Never	6,380 (87.69)	4,184 (88.49)	2,196 (86.19)
Quit	97 (1.33)	37 (0.78)	60 (2.35)
Not asked, missing	510 (7.01)	326 (6.90)	184 (7.22)
Depression ICD10 code	999 (13.73)	624 (13.20)	375 (14.72)
Comorbidity count			
0–1 diagnosis	1,934 (26.58)	1,280 (27.07)	654 (25.67)
2–3 diagnoses	3,663 (50.34)	2,384 (50.42)	1,279 (50.20)
>3 diagnoses	1,679 (23.08)	1,064 (22.50)	615 (24.14)
Insurance type			
Commercial or others	3,308 (45.46)	2,160 (45.69)	1,148 (45.05)
Medicare	3,183 (43.75)	2,069 (43.76)	1,114 (43.72)
Medicaid	785 (10.79)	499 (10.55)	286 (11.22)
Scheduled with a nurse practitioner	4,516 (62.07)	2,974 (62.90)	1,542 (60.52)
Scheduling lag category, days			
≤7	1,651 (22.69)	528 (11.17)	1,123 (44.07)
8–30	1,590 (21.85)	684 (14.47)	906 (35.56)
31–90	1,554 (21.36)	1,190 (25.17)	364 (14.29)
≥91	2,481 (34.10)	2,326 (49.20)	155 (6.08)

BMI, body mass index; COVID, coronavirus disease; ED, emergency department; HbA1c, glycated hemoglobin; ICD10, the 10th revision of the International Statistical Classification of Diseases and Related Health Problems.

Among 2,235 patients, 1,102 patients were seen in both periods, 811 patients only during pre-COVID, and 322 only during COVID. In this dataset, each patient scheduled 3.26 appointments on average (ranged 1–18, standard deviation [SD] 2.21), where during pre-COVID each patient scheduled 2.47 appointments on average (range 1–14, SD 1.56) and during COVID the average scheduled appointments was 1.79 (ranged 1–9, SD 0.98). Overall, 49.53% of the included patients were female, and 48.72% Black with 40.94% White. Majority of the included patients (83.89%) had an activated MyChart account.

More patients during COVID had an activated MyChart account compared with pre-COVID (88.24% vs. 81.59%). During COVID, a higher percentage of appointments were with A1c drawn >6 months, missing BMI, missing smoking status, scheduling within a week.

### PREDICTORS OF APPOINTMENT NO-SHOW

#### Unadjusted model

*Table 2* shows the unadjusted ORs of appointment no-shows by time periods and the *p*-value for the interaction between a variable and time periods. Majority of the unadjusted results were similar in direction across both periods if comparing qualitatively. Protective factors against appointment no-shows included older age (vs. <46 years), White or other race (vs. Black), controlled HbA1c (compared with uncontrolled HbA1c), use more than metformin but no insulin (vs. any insulin), patient portal account activation (vs. no), and being married (vs. single, widowed, or unknown). Risk factors of appointment no-shows across both periods were missing BMI (compared with BMI ≤24.9), ED visit 12 months prior (vs. no) and hospitalization 12 months prior (vs. no), never use alcohol (vs. active alcohol use), having a Medicare or Medicaid (vs. commercial or other), home distance ≤30 miles (vs. >30 miles), and longer scheduling lag (vs. ≤7 days).

**Table 2.** Unadjusted Odds Ratios of Appointment No-Shows and *p*-Value for Interaction for COVID Period

VARIABLE	PRE-COVID		COVID		***p***-VALUE FOR INTERACTION
	ORs	95% CI	ORs	95% CI	
Basic characteristics					
Age (ref <46 years old)					
46–60	0.71	(0.50–1.01)	0.62	(0.41–0.94)	0.879
61–75	0.45	(0.32–0.64)	0.37	(0.24–0.57)	0.604
>75	0.60	(0.38–0.95)	0.40	(0.22–0.73)	0.290
Female (ref: male)	1.65	(1.31–2.06)	1.27	(0.96–1.69)	0.130
Race (ref: black)					
White	0.28	(0.21–0.36)	0.42	(0.31–0.59)	0.126
Others	0.49	(0.34–0.72)	0.50	(0.29–0.87)	0.899
Ethnicity as Hispanic (ref: not Hispanic)	0.89	(0.47–1.68)	0.89	(0.37–2.11)	0.848
DM related					
HbA1c (ref: uncontrolled)					
Controlled	0.55	(0.43–0.69)	0.56	(0.41–0.78)	0.709
Missing	0.95	(0.71–1.27)	1.39	(0.95–2.04)	0.294
HbA1c drawn month (ref: 1–3 months ago)					
Missing	1.19	(0.88–1.61)	2.51	(1.64–3.84)	0.015
4–6	1.05	(0.84–1.32)	1.85	(1.29–2.65)	0.002
7–9	0.84	(0.62–1.15)	1.87	(1.23–2.83)	0.001
10–12	1.04	(0.67–1.61)	1.71	(0.96–3.04)	0.219
BMI (ref: BMI ≤24.9)					
Overweight	0.75	(0.52–1.08)	0.81	(0.48–1.34)	0.946
Class 1 obesity	0.97	(0.67–1.40)	1.02	(0.63–1.67)	0.951
Class 2 obesity	0.76	(0.51–1.14)	1.01	(0.59–1.71)	0.295
Class 3 obesity	1.21	(0.80–1.83)	1.08	(0.62–1.86)	0.650
Missing	46.12	(9.98–212.99)	2.47	(1.28–4.77)	<0.001
Medication (ref: any insulin)					
No medication	0.89	0.57–1.39	0.83	0.53–1.31	0.594
Metformin only	0.71	0.43–1.19	0.19	0.36–1.87	0.552
More than metformin but no insulin	0.65	0.47–0.91	0.38	0.22–0.68	0.105
Diabetes complications (ref: no complications ICD10)	1.38	(0.96–1.97)	1.34	(0.81–2.22)	0.665
Health utilization					
ED visit in prior 12 months (ref: no)	1.93	(1.53–2.43)	2.12	(1.56–2.87)	0.906
Hospitalization in prior 12 months (ref: no)	1.48	(1.15–1.90)	1.72	(1.25–3.37)	0.671
Active patient portal Account (ref: inactive)	0.37	(0.28–0.50)	0.41	(0.28–0.60)	0.482
Other health behavior					
Smoking status (ref: active)					
Never	0.29	(0.20–0.41)	0.68	(0.43–1.09)	0.009
Quit	0.38	(0.26–0.55)	0.61	(0.37–1.00)	0.188
Not asked, missing	1.89	(0.63–5.67)	2.37	(1.11–5.05)	0.911
Alcohol status (ref: active)					
Never	1.45	(1.14–1.84)	1.46	(1.06–2.01)	0.807
Quit	1.34	(0.72–2.51)	0.80	(0.43–1.48)	0.300
Not asked	1.68	(1.00–2.83)	2.15	(1.21–3.83)	0.630
Recreational drug status (ref: active)					
Never	0.33	(0.20–0.56)	0.60	(0.32–1.12)	0.161
Quit	1.61	(0.56–4.65)	1.22	(0.45–3.32)	0.879
Not asked	0.48	(0.25–0.90)	1.01	(1.03–2.17)	0.213
Comorbidities					
Had a depression ICD10 (ref: no)	1.31	(0.95–1.79)	1.49	(1.03–2.17)	0.515
Comorbidity count (ref: 0–1 diagnosis)					
2–3 diagnoses	1.16	(0.90–1.49)	0.78	(0.56–1.09)	0.105
>3 diagnoses	1.16	(0.85–1.57)	0.95	(0.64–1.39)	0.389
Other SDOHs					
Insurance (ref: commercial and others)					
Medicare	1.42	(1.12–1.80)	1.59	(1.16–2.19)	0.656
Medicaid	4.07	(2.87–5.78)	3.26	(2.09–5.07)	0.857
Married (ref: single, unknown)	0.43	(0.34–0.54)	0.44	(0.33–0.59)	0.767
English as preferred language (ref: no)	0.72	(0.36–1.42)	0.46	(0.21–1.00)	0.143
Family member as emergency contact (ref: no)	0.86	(0.57–1.29)	0.78	(0.47–1.29)	0.735
Home distance ≤30 miles (ref: >30 miles)	2.69	(1.85–3.90)	1.8	(1.13–2.86)	0.342
Health provider factors					
Scheduled with a nurse practitioner (ref: physician)	1.25	(1.03–1.53)	2.36	(1.71–3.27)	0.001
Health system factor-scheduling lag					
Scheduling lag (ref: ≤7 days)					
8–30	1.51	1.03–2.21	1.52	1.11–2.10	0.915
31–90	2.32	1.62–3.33	1.39	0.91–2.13	0.228
≥91	2.09	1.46–2.98	2.46	1.43–4.25	0.117
Health system factor-visit factors					
Telehealth as delivery mode (ref: in-person)	—		0.43	(0.32–0.57)	—
Time period					
COVID (ref: early COVID)	—		0.92	(0.70–1.21)	—

CI, confidence interval; DM, diabetes mellitus; ORs, odds ratios; SDOHs, social determinants of health.

Several factors were shown as risk factors only during COVID and not during pre-COVID: missing an HbA1c on file or an HbA1c was drawn 4–9 months before this appointment (vs. drawn 1–3 months ago), missing smoking status (vs. active smoker), and having a depression ICD10 (vs. no). Telehealth was a protective factor against appointment no-shows during COVID.

The interaction terms with COVID periods (i.e., pre-COVID vs. COVID) were significant for the following variables: months when HbA1c was drawn before this visit, missing BMI, never smoking, and scheduled with a nurse practitioner (NP). Most of the variables were in same directions but with different magnitude across both periods. For instance, compared with HbA1c drawn within 1–3 months before the scheduled appointments, the HbA1c on file was drawn 4–6 months (pre-COVID vs. COVID ORs: 1.19 vs. 2.51, *p*-value for interaction: 0.015), and was much stronger risk of appointment no-shows during COVID than pre-COVID. Only the HbA1c on file drawn 7–9 months before this visit had different direction across COVID periods—it was protective against appointment no-shows during pre-COVID but a risk factor during COVID (pre-COVID vs. COVID ORs: 0.84 vs. 1.87, *p*-value for interaction: 0.001).

#### Adjusted model

Results of adjusted odds ratios (aORs) of appointment no-shows by COVID are shown in *Table 3*. Similar to unadjusted model, adjusted results were similar in direction across periods with moderate magnitude in the adjusted model.

**Table 3.** Adjusted Odds Ratios of Appointment No-Show for COVID Period

VARIABLE	PRE-COVID			COVID		
	aORs	95% CI	***p***	aORs		95% CI	***p***
Basic characteristics						
Age (ref <46 years old)						
46–60	0.73	0.50–1.06	0.100	0.83		0.54–1.28	0.405
61–75	0.49	0.33–0.73	0.001	0.39		0.24–0.63	0.000
>75	0.69	0.40–1.18	0.171	0.40		0.21–0.79	0.008
Female (ref: male)	1.20	0.94–1.53	0.151	—			
Race (ref: black)							
White	0.38	0.28–0.50	0.000	0.67		0.47–0.96	0.029
Others	0.70	0.46–1.06	0.091	0.87		0.48–1.56	0.632
Ethnicity as Hispanic (ref: not Hispanic)	—			—			
DM related							
HbA1c (ref: uncontrolled)							
Controlled	0.62	0.48–0.80	0.000	0.72		0.51–1.02	0.061
Missing	1.15	0.83–1.58	0.398	2.30		1.39–3.79	0.001
HbA1c drawn month (ref: 1–3 months ago)							
Missing	—				Omitted		
4–6					1.92	1.31–2.80	0.001
7–9					2.26	1.46–3.49	0.000
10–12					1.68	0.92–3.08	0.093
BMI (ref: BMI ≤24.9)							
Overweight	0.87	0.59–1.28	0.481	0.89		0.53–1.52	0.678
Class 1 obesity	1.09	0.74–1.61	0.664	1.13		0.67–189	0.655
Class 2 obesity	0.70	0.46–1.07	0.101	0.86		0.49–1.50	0.599
Class 3 obesity	0.97	0.62–1.51	0.883	0.88		0.49–1.56	0.650
Missing	36.96	6.58–207.51	0.000	2.47		1.05–5.78	0.037
Medication (ref: any insulin)							
No medication	1.17	0.73–1.87	0.524	1.10		0.67–1.80	0.703
Metformin only	0.88	0.51–1.53	0.654	1.22		0.84–1.98	0.648
More than metformin but no insulin	0.92	0.64–1.31	0.628	0.55		0.38–0.84	0.049
DM complications (ref: no complications ICD10)	—				—		
Health utilization							
ED visit in prior 12 months (ref: no)	1.56	1.15–2.12	0.004	1.53		1.01–2.31	0.046
Hospitalization in prior 12 months (ref: no)	1.12	0.81–1.54	0.505	1.29		0.84–1.98	0.253
Active patient portal account (ref: inactive)	0.54	0.40–0.73	0.000	0.57		0.38–0.84	0.005
Other health behavior							
Smoking status (ref: active)							
Never	0.40	0.27–0.59	0.000	1.16		0.71–1.89	0.566
Quit	0.53	0.36–0.78	0.001	0.99		0.59–1.67	0.979
Not asked, missing	0.34	0.07–1.64	0.180	1.87		0.64–5.48	0.256
Alcohol status (ref: active)							
Never	1.23	0.95–1.59	0.111	1.14		0.82–1.60	0.438
Quit	0.93	0.48–1.79	0.818	0.61		0.32–1.17	0.140
Not asked	0.87	0.40–1.88	0.719	1.17		0.50–2.76	0.718
Recreational drug status (ref: active)							
Never	0.61	0.35–1.07	0.085	—			
Quit	2.03	0.67–6.19	0.212				
Not asked	1.15	0.52–2.54	0.724				
Comorbidities							
Had a depression ICD10 (ref: no)	—				1.45	0.98–2.15	0.062
Other SDOHs							
Insurance: (ref: commercial and others)							
Medicare	1.08	0.81–1.45	0.604	1.57		1.06–2.32	0.025
Medicaid	1.68	1.14–2.48	0.009	1.42		0.89–2.26	0.140
Married (ref: single, unknown)	0.82	0.63–1.06	0.125	0.76		0.55–1.06	0.110
Home distance ≤30 miles (ref: >30 miles)	1.63	1.11–2.42	0.014	1.15		0.70–1.88	0.589
Health provider factors							
Scheduled with a NP (ref: physician)	1.36	1.08–1.71	0.009	2.20		1.56–3.11	0.000
Health system factor-scheduling lag							
Scheduling lag (ref: ≤7 days)							
8–30	1.34	0.92–1.97	0.131	1.45		1.05–2.00	0.025
31–90	2.24	1.56–3.22	0.000	1.04		0.67–1.62	0.866
≥91	2.96	2.04–4.29	0.000	1.85	1.04–3.30	0.037	
Health system factor-visit factors							
Telehealth as delivery mode (ref: in-person)	—				0.40	0.29–0.57	0.000

aORs, adjusted odds ratios.

Comparing adjusted models across periods, 61–75 years old, being White race, having an activated patient portal account, were protective factors against appointment no-shows, while missing BMI on file, ED visits 12 months before the scheduled visit, scheduling with an NP, and scheduling lag ≥91 days were predictive of appointment no-shows.

### INTERACTION OF PREDICTOR VARIABLES WITH HEALTH CARE DELIVERY MODES

The stratified unadjusted ORs of appointment no-shows by health care delivery modes (in-person vs. telehealth visits) are presented in *Table 4*. When testing for interaction between the predictor variables and the health care delivery modes during COVID, none of the interactions was significant. We observed some trends in the magnitude of the OR. Being Hispanic was protective against appointment no-shows for telehealth visits (OR: 0.37) but not for in-person visits (OR: 1.84). We also found longer scheduling lags, where scheduling between 1 and 3 months, was as protective against appointment no-shows in telehealth visits (OR: 0.88), but a risk factor in in-person visits (OR: 1.48) and the same in scheduling more than 3 months ago (telehealth vs. in-person ORs: 0.65 vs. 2.18). Moreover, we found that having a missing HbA1c on file had a higher odds of appointment no-show in in-person visits (OR: 1.89) compared with telehealth visits (OR: 1.11). A similar trend was also observed in the variable when HbA1c was drawn 10–12 months before this appointment (telehealth vs. in-person ORs: 1.08 vs. 2.20).

**Table 4.** Stratified Unadjusted Odds Ratio of Appointment No-Show by Health Care Delivery Mode During COVID

VARIABLE	TELEHEALTH		IN-PERSON	
	UNADJUSTED		UNADJUSTED	
	OR (95% CI)	***p***	OR (95% CI)	***p***
Basic characteristics				
Age: (ref <46 years old)				
46–60	0.59 (0.37–0.94)	0.027	0.75 (0.36–1.53)	0.425
61–75	0.35 (0.21–0.57)	0.000	0.42 (0.20–0.85)	0.016
>75	0.37 (0.18–0.73)	0.004	0.47 (0.19–1.17)	0.106
Female (ref: male)	1.30 (0.93–1.82)	0.123	1.16 (0.74–1.83)	0.506
Race (ref: black)				
White	0.41 (0.28–0.61)	0.000	0.50 (0.30–0.85)	0.010
Others	0.37 (0.18–0.77)	0.008	0.83 (0.38–1.80)	0.638
Ethnicity (ref: not Hispanic)	0.37 (0.86–1.61)	0.187	1.84 (0.64–5.28)	0.260
DM related				
HbA1c (ref: uncontrolled)				
Controlled	0.55 (0.37–0.82)	0.003	0.62 (0.36–1.06)	0.083
Missing	1.11 (0.69–1.80)	0.656	1.89 (1.06–3.37)	0.031
HbA1c month (ref: 1–3 months)				
Missing	2.11 (1.24–3.58)	0.006	2.91 (1.52–5.56)	0.001
4–6	2.10 (1.38–3.21)	0.001	1.36 (0.70–2.64)	0.361
7–9	2.05 (1.25–3.39)	0.005	1.37 (0.69–2.73)	0.363
10–12	1.08 (0.46–2.54)	0.854	2.20 (1.01–4.78)	0.047
BMI (ref: BMI ≤24.9)				
Overweight	0.71 (0.38–1.30)	0.266	0.88 (0.41–1.92)	0.752
Class 1 obesity	1.04 (0.59–1.84)	0.885	0.98 (0.45–2.14)	0.956
Class 2 obesity	1.06 (0.57–1.94)	0.862	0.83 (0.35–1.96)	0.667
Class 3 obesity	1.25 (0.68–2.31)	0.468	0.74 (0.28–1.99)	0.554
Missing	1.52 (0.70–3.31)	0.294	Empty	
Diabetes medication (ref: insulin)				
No medication	0.85 (0.50–1.47)	0.569	0.77 (0.37–1.59)	0.479
Metformin only	0.82 (0.30–2.23)	0.699	0.72 (0.21–2.52)	0.608
More than metformin but no insulin	0.35 (0.17–0.73)	0.005	0.44 (0.19–1.00)	0.050
Diabetes complications: (ref: no complications ICD10)	1.51 (0.80–2.86)	0.205	1.11 (0.52–2.35)	0.787
Health utilization				
ED visit in prior 12 months (ref: no)	1.99 (1.38–2.85)	0.000	2.21 (1.37–3.56)	0.001
Hospitalization in prior 12 months (ref: no)	1.68 (1.16–2.46)	0.007	1.70 (1.02–2.82)	0.040
Patient portal account activated (ref: no)	0.43 (0.28–0.67)	0.000	0.50 (0.29–0.86)	0.012
Other health behavior				
Smoker status (ref: active)				
Never	0.77 (0.44–1.36)	0.375	0.63 (0.32–1.26)	0.193
Quit	0.76 (0.42–1.39)	0.377	0.48 (0.23–1.03)	0.059
Not asked, missing	2.15 (0.88–5.28)	0.095	3.62 (0.89–14.77)	0.072
Alcohol status (ref: active)				
Never	1.42 (0.97–2.06)	0.068	1.38 (0.83–2.32)	0.217
Quit	0.77 (0.37–1.61)	0.493	0.81 (0.29–2.27)	0.690
Not asked	1.91 (0.97–3.75)	0.059	2.46 (0.97–6.22)	0.058
Drug status (ref: active)				
Never	0.70 (0.32–1.51)	0.359	0.55 (0.23–1.35)	0.192
Quit	0.79 (0.20–3.04)	0.728	1.80 (0.48–6.77)	0.385
Not asked	0.98 (0.38–2.50)	0.966	1.22 (0.40–3.69)	0.722
Comorbidities				
Depression (ref: no ICD10)	1.39 (0.89 = 2.15)	0.145	1.54 (0.76–2.72)	0.141
Comorbidity count (ref: 0–1 diagnosis)				
2–3 diagnoses	0.92 (0.63–1.37)	0.694	0.53 (0.30–0.91)	0.022
>3 diagnoses	0.85 (0.53–1.36)	0.492	1.03 (0.57–1.85)	0.923
Other SDOHs				
Insurance (ref: commercial and others)				
Medicare	1.56 (1.08–2.26)	0.018	1.46 (0.86–2.46)	0.161
Medicaid	2.42 (1.43–4.08)	0.001	4.24 (1.97–9.11)	0.000
Marital status: married (ref: single)	0.40 (0.28–0.57)	0.000	0.59 (0.38–0.93)	0.023
Preferred language as English (ref: no)	0.52 (0.20–1.37)	0.184	0.54 (0.19–1.57)	0.260
Contact as family (ref: no)	0.94 (0.50–1.76)	0.837	0.68 (0.33–1.38)	0.284
Distance from home to clinic ≤30 miles (ref: >30 miles)	1.71 (1.00–2.94)	0.051	1.78 (0.82–3.85)	0.146
Health provider factors				
Scheduled with a nurse practitioner (ref: physician)	2.72 (1.82–4.07)	0.000	1.86 (1.15–3.00)	0.011
Health system factor-scheduling lag				
Scheduling lag (ref: ≤7 days)				
8–30	1.34 (0.95–1.90)	0.097	1.97 (1.02–3.82)	0.043
31–90	0.88 (0.48–1.59)	0.671	1.48 (0.75–2.90)	0.259
≥91	0.65 (0.19–2.20)	0.489	2.18 (1.08–4.42)	0.030

## Discussion

This study examined a comprehensive set of multilevel predictors of appointment no-shows among adults with T2DM comparing pre-COVID and COVID period based on the most current systematic review.^16^ While previous studies focused on in-person visits, our study findings add to the current literature to examine the impact of COVID and telehealth implementation to predictors of appointment no-shows among people with T2DM.

We found that missing HbA1c and BMI or an HbA1c drawn longer than the recommended 3-month interval^8^ was a risk factor of appointment no-shows during COVID. As measuring BMI is a standard procedure for in-person visits, less than 0.5% of encounters in our dataset had a missing BMI during pre-COVID, which could result in an unstable estimate of the OR due to insufficient sample size. Whether or not limited in-person activities during COVID led to worse glycemic control or compromised other diabetes quality indicators remained unanswered as current literature had conflicting results.^27–29^ However, limited in-person activities do not equal to disengagement from health care. For example, diabetes medication prescribing stayed the same level during COVID as pre-COVID in a national cohort.^28^ It would be important to identify and implement effective strategies to ensure a patient with T2DM are engaged in health care regardless of either delivery method (in-person or telehealth). For example, a recent systematic review revealed that using patient portal was essential for patients to stay engaged in their care, including communicating with their providers.^30^

In fact, having an active patient portal account decreased more than 50% of the odds of appointment no-shows during both periods. More patients were introduced to patient portal during COVID than pre-COVID (88% vs. 82%) in our study due to how telehealth visits set up in the diabetes clinics. Still, more than 10% of patients did not have an active account. Among people without an activated patient portal account, more than 70% were >60 years old and non-White race (data not shown). White populations with higher education levels are repeatedly more likely to adopt using a patient portal.^31,32^ These findings highlight the need for designing a patient portal for patients with diverse backgrounds in mind (e.g., older patients, lower education levels) to use a patient portal to further engage in their diabetes care.

The implementation of telehealth reduced appointment no-shows compared with in-person visits during COVID (aOR: 0.40, 95% confidence interval 0.29–0.57, *p* < 0.001) in this study. Telehealth seems to provide an opportunity to democratize diabetes care to racial or social minority populations^33^ by addressing some of the barriers.^18,19^ However, telehealth visits require access to reliable internet and technological devices. It is worth noting that only 78.0% of the household in Baltimore subscribed to Broadband internet compared with 88.5% in Maryland.^34^ Additionally, people with limited English proficiency, older adults, and people from racial minorities are less likely to use video visits compared with phone visits in primary care because of limited access to broadband internet and/or digital devices.^35,36^ Indeed, the National Academies of Sciences, Engineering, and Medicine warns that the digital divide might exacerbate health care disparities.^37,38^ These results underscore the importance of addressing SDOHs in bridging the gap in using technology/telehealth visits in diabetes care, especially for racial and social minorities.

When comparing predictors of appointment no-shows during COVID by health care delivery modes (i.e., in-person vs. telehealth visits), we did not find any statistically significant results. Instead, we found longer scheduling lag for telehealth visits tended to have a OR <1 compared with in-person visits. This trend could be explained by the efforts of outreaching at the health care provider and system level. Providers and staff were required to verify patients' locations to provide care based on individual state regulations^39^, and those calls might have served as a reminder of the scheduled appointment. Additionally, providers would reach out to patients to verify technological problems if patients were not online from our clinical experience. Future research is warranted to better understand if predictors of appointment no-shows differ between in-person and telehealth visits.

In our study, older age and White race were protective against appointment no-shows in both unadjusted and adjusted models during both periods. The finding is consistent with prior research in the United States where being racial minorities with diabetes was associated with higher appointment no-shows.^40,41^ More than half of the participants in the analysis dataset were racial minorities.^42^ Race in these studies might have been a proxy indicator of SDOHs (e.g., education attainment or income level).^43,44^ For instance, comparing Baltimore city with 62.3% Black, where the clinics are located, to Maryland with 31.4% Black, 2020 Census revealed that in Baltimore more residents ≥25 years had a high school degree or less (41.9% vs. 33.6%) and the median household income was lower ($52,164 vs. $87,063 in 2020$).^34^ To promote appointment keeping and reduce health disparities, future research should explore the intersectionality of race with SDOHs and patient engagement in diabetes care.

Additionally, the finding suggests the need for better documentation of SDOHs in EHR. ADA recommends routine screening for SDOHs as they contribute to medical and psychosocial outcomes and further affect one's ability to manage their diabetes.^8^ Yet, these factors are either unmeasured or poorly documented in the EHR or were sparsely mentioned in free texts, in which a more sophisticated data extraction method is required.^45,46^

NPs have been an integral workforce in health care and provided high quality of care.^47^ According to a secondary analysis of the Consumer Assessment of Health care Providers and Systems survey, patients rated NPs significantly higher in satisfaction than other health care providers, including physicians.^48^ However, contrary to those facts, our study found that scheduling with an NP had 36–120% of increased odds of appointment no-shows in comparison with a physician during both periods. In our diabetes practice, new patients with diabetes are required to see a physician first. Since the reasons behind appointment no-shows might be different between new and established patients due to rapport, we excluded all new patient appointments in the analyses. Additionally, more than 60% of the included appointments were scheduled with a NP. To this end, our finding may be an artifact of sampling bias, not an evaluation on different types of providers. A qualitative inquiry to both providers and patients is warranted to understand this phenomenon and the care transition between team members.

Our study is not without limitations. First, unmeasured predictors of appointment no-shows, such as financial hardship, change of insurance coverage during COVID, access to internet or technical devices, or COVID-related health impact were not captured in EHR. This might lead to uncontrolled biases to our comprehensive model. Additionally, this study was not powered for the interaction analyses. There were more telehealth visits scheduled during the first 9 months of COVID in 2020 compared with current clinical practices. This might potentially over or underestimate the OR in the results. However, the COVID period included in the study were relatively homogeneous compared with calendar year 2021 as COVID vaccine was approved under emergency use authorization on December 11, 2020^49^ and the massive vaccine roll out to general populations did not happen until 2021.^50^

## Conclusions

Keeping regular diabetes appointment is essential in diabetes care for better health outcomes.^8^ Since the COVID pandemic, diabetes care and other chronic care delivery mode has rapidly expanded to include telehealth visits. We found that using patient portal and telehealth visits reduced the odds of appointment no-shows in the context of diabetes care. Future studies are needed to address SDOHs, including access to internet, to provide personalized quality care, and to further reduce health disparities among adults with T2DM.

## Supplementary Material

Supplemental data
