# Supplementary material for: Predictors of Follow-Up Appointment No-Shows Before and During COVID Among Adults with Type 2 Diabetes
Source: Telemed J E Health. 2023 Jun 1;29(6):851–65. doi: 10.1089/tmj.2022.0377 (PMC10277979; doi:10.1089/tmj.2022.0377)
Supplement: Supplemental data [file Suppl_TableS1.docx]

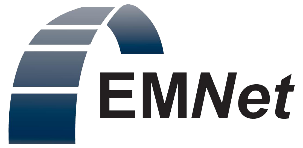

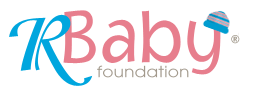

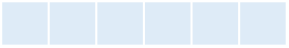

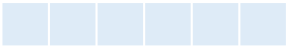


**Department Inventory**

**National Emergency**

**4) Please indicate the total number of patient visits at your ED and the 12-month reporting period to which they apply:**

**3) In 2019, was your ED open: a. 24 hours/day, 7 days/week?**  YES NO **b. 365 days/year ?**  YES NO

If **NO** to either question, please explain: ___________________________________________________________

# ED VISITS

**5) Please indicate the approximate number of ED visits by children (e.g., age <18):**

If your ED uses another age to distinguish between children and adults (e.g., age 21 years), and it’s difficult to obtain data for age

<18, please respond according to your ED’s cut-off.

# CHILD ED VISITS

**Specify cut-off if other than <18 years**: age < _____ years

Thank you! Please contact us at [emnet@partners.org](mailto:emnet@partners.org) or at **617-724-4069** with any questions.

*****additional

info on

other side

**1) What is the name of your hospital/ED? ______________________________________________________________**

**2) What is the mailing address of your hospital/ED? _____________________________________________________**

This survey is funded by the R Baby Foundation ([www.rbabyfoundation.org](http://www.rbabyfoundation.org))

Please find below questions that apply to the year **2019** (either fiscal or calendar year is acceptable). If you are unable to quickly find the precise answer, we welcome your best estimate.

The results from this national survey will be used to perform health services research on U.S. emergency care, and to update our existing app, *EMNet findERnow*. We hope that you can help us with this short/simple survey as we work to bring accurate, beneficial ED information directly to those in need.

Please send your answers to us in the attached, **pre-paid envelope**. If you prefer, we also welcome your responses by email ([emnet@partners.org](mailto:emnet@partners.org)) or by fax to **617-724-4050**. Another option is to complete this form online at: <https://is.gd/findERnow2019>.

We would like to know the following about your ED:

**9) Does your hospital/ED *provide* telemedicine services *out* for the evaluation of patients in other EDs? ***

YES NO Not sure

**8) Does your ED *receive* telemedicine services for patient evaluation from another facility or outside entity? ***

YES

NO

**Does your ED utilize telemedicine for:** *(check all that apply)*

🞎 Pediatrics 🞎 Psychiatry 🞎 Dermatology 🞎 Transfer coordination

🞎 Stroke/neuro 🞎 Trauma 🞎 Radiology 🞎 Disaster preparedness

🞎 Other: _______________________________________________________

**7) Do you have identified coordinators for pediatric emergency care in your ED? ***

YES

NO

**Please specify:** *(check all that apply)*

🞎 Physician coordinator(s) 🞎 Nurse coordinator(s)

🞎 Other coordinator (e.g., PA, administrator): __________________________

**6)** For EDs that regularly treat adults: **Does your ED have a dedicated area for children only (e.g., dedicated beds)?**

YES NO Not applicable (e.g., children’s hospital)

**Reporting period:** From ___/___ to ___/___

mm yy mm yy

Provide telemedicine services out to

- **If your hospital/ED *provides* telemedicine *out*:**


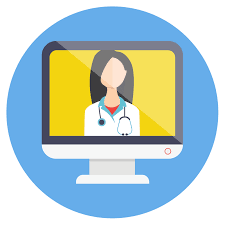


Clinicians in your hospital/ED

Patients in other EDs


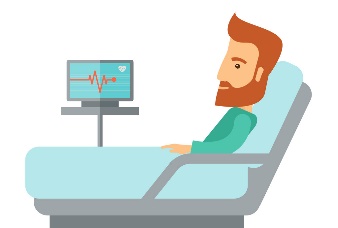


**Question 7:** A pediatric emergency care coordinator (PECC) is someone who manages pediatric care in the ED and who helps educate other ED staff on pediatric emergency care. PECC roles can vary, and some EDs refer to these individuals with different titles (e.g., Pediatric Champion).

**Questions 8 & 9:** Please see the below diagram:

***Additional Information**

Receive telemedicine services from

services from

- **If your ED *receives* telemedicine:**

Patients in your ED

Clinicians from another facility or outside entity


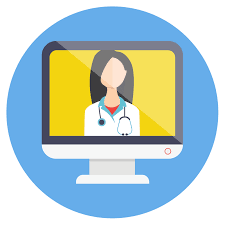

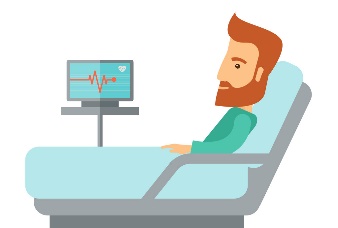


**We welcome your comments and suggestions! Please feel free to write in the space below, or to contact us**:

Carlos Camargo, MD, DrPH Phone: 617-724-4069

Massachusetts General Hospital Fax: 617-724-4050

125 Nashua Street, Suite 920 [emnet@partners.org](mailto:emnet@partners.org)

Boston, MA 02114 [www.emnet-usa.org](http://www.emnet-usa.org)

____________________________________________________________________________________________________________________________________________________________________________________________________________________________________________________________________________________________________________________________________________________________________________________________________________________________________________________________________________________________________________________________________________________________________________________

**Who completed this survey?** We may want to reach out to you to learn more about your ED. Please complete the fields that you feel comfortable sharing. We will not share your individual contact information.

Name __________________________________________________________________________

Position (e.g., ED Director) _________________________________________________________

Email __________________________________________________________________________

Phone __________________________________________________________________________

Receive telemedicine

services from

- **If your ED *receives* telemedicine:**

Patients in your ED

Clinicians from another facility or outside entity


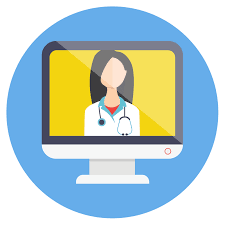

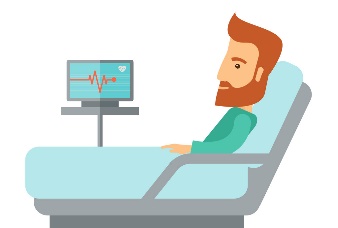


Provide telemedicine services out to

- **If your hospital/ED *provides* telemedicine *out*:**


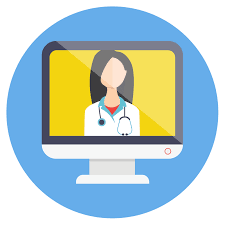


Clinicians in your hospital/ED

Patients in other EDs


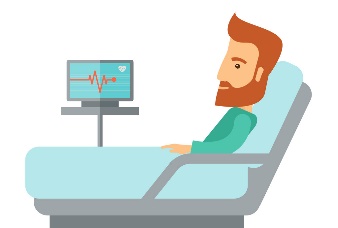


***Additional Information**

**Question 7:** A pediatric emergency care coordinator (PECC) is someone who manages pediatric care in the ED and who helps educate other ED staff on pediatric emergency care. PECC roles can vary, and some EDs refer to these individuals with different titles (e.g., Pediatric Champion).

**Questions 8 & 9:** Please see the below diagram:
